# Supplementary material for: Microendoscopic Posterior Decompression for Treating Thoracic Myelopathy Caused by Ossification of the Ligamentum Flavum: Case Series
Source: Medicina (Kaunas). 2020 Dec 10;56(12):684. doi: 10.3390/medicina56120684 (PMC7763969; doi:10.3390/medicina56120684)
Supplement: Supplementary file 1 [file medicina-56-00684-s001.pdf]

Supplementary Table 1. Summary of 7 cases of thoracic OLF treated by conventional open surgery.

Patient	Age	Sex	Level	Type (Axial CT)	Type (Sagittal MRI)	Intensity change (MRI)	Approach	Operation time (minutes)	Resection	Pre-mJOA	Post-mJOA	Recovery Rate (%)	Follow up month	Complication	Combined spinal lesions
1	72	F	T11/12/L1	bilateral	round	+	decompression alone	112	Complete	7	7	0	52	–	C+ post / L+ post
2	74	M	T1/2/3	bilateral	round	+	decompression alone	155	Complete	6	6	0	3	SSI	L+ post
3	59	M	T2/3	bilateral	beak	+	decompression alone	147	Complete	6	6	0	42	–	N
4	75	M	T10/11	bilateral	beak	+	decompression alone	76	Complete	5	5	0	42	–	C-
5	66	M	T11/12	bilateral	round	−	decompression alone	82	Complete	8	9	33	3	–	L+ post
6	79	M	T10/11	bilateral	round	−	decompression with fusion	83	Complete	4	4	0	2	–	L+ post
7	69	F	T11/12	bilateral	round	+	decompression with fusion	162	Complete	8	8	0	15	–	L-

CT: computed tomography, MRI: magnetic resonance image, mJOA: modified Japanese Orthopaedic Association, M: male, F: female, –: no intensity change is present, +: intensity change present, –: no complication, SSI surgical site infection, C+ cervical canal stenosis for operation, C- cervical canal stenosis for observation, L+ lumber canal stenosis for operation, L- lumber canal stenosis for observation, N no operation, post means operation before the thoracic OLF, pre means operation after the thoracic OLF.
